# Relative difference in muscle strength between patients with knee osteoarthritis and healthy controls when tested bilaterally and joint-inclusive: an exploratory cross-sectional study

**DOI:** 10.1186/s12891-019-2957-6

**Published:** 2019-12-09

**Authors:** K. Vårbakken, H. Lorås, K. G. Nilsson, M. Engdal, A. K. Stensdotter

**Affiliations:** 1 Norwegian University of Science and Technology, Trondheim, Norway; 2 Department of Physical Education and Sport Science, Nord University, Levanger, Norway; 3 Umea University, Surgical and Perioperative Sciences, Umea, Sweden; 4 Department of Physiotherapy, Clinic of Clinical Services, Trondheim University Hospital, Trondheim, Norway

**Keywords:** Osteoarthritis, knee, Healthy volunteers, Muscle strength, Muscle strength dynamometer, Primary health care, Secondary care, Cross-sectional studies [Mesh terms], Exploratory studies [text-word]

## Abstract

**Background:**

To improve the goal-directedness of strength exercises for patients with knee osteoarthritis (KOA), physical rehabilitation specialists need to know which muscle-groups are most substantially weakened across the kinetic chain of both lower extremities. The purpose was to improve the knowledge base for strength exercise therapy. The objective was to explore the relative differences in muscle strength in the main directions bilaterally across the hip, knee, and ankle joints between patients with light-to-moderate symptomatic and radiographic KOA and people without knee complaints.

**Methods:**

The design was an exploratory, patient vs. healthy control, and cross-sectional study in primary/secondary care. Twenty-eight patients with mild to moderate KOA (18 females, mean age 61) and 31 matched healthy participants (16 females, mean age 55), participated. Peak strength was tested concentrically or isometrically in all main directions for the hip, knee, and ankle joints bilaterally, and compared between groups. Strength was measured by a Biodex Dynamometer or a Commander II Muscle Tester (Hand-Held Dynamometer). Effect sizes (ES) as Cohen’s *d* were applied to scale and rank the difference in strength measures between the groups. Adjustment for age was performed by analysis of covariance.

**Results:**

The most substantial muscle weaknesses were found for ankle eversion and hip external and internal rotation in the involved leg in the KOA-group compared to the control-group (ES [95% CI] −0.73 [−1.26,-0.20], − 0.74 [−1.26,-0.21], −0.71 [−1.24,-0.19], respectively; *p* < 0.01). Additionally, smaller but still significant moderate muscle weaknesses were indicated in four joint–strength directions: the involved leg’s ankle inversion, ankle plantar flexion, and knee extension, as well as the uninvolved leg’s ankle dorsal flexion (*p* < 0.05). There was no significant difference for 17 of 24 tests.

**Conclusions:**

For patients with KOA between 45 and 70 years old, these explorative findings indicate the most substantial weaknesses of the involved leg to be in ankle and hip muscles with main actions in the frontal and transverse plane in the kinetic chain of importance during gait. Slightly less substantial, they also indicate important weakness of the knee extensor muscles. Confirmatory studies are needed to further validate these exploratory findings.

## Background

Osteoarthritis (OA) is a leading cause of pain and disability worldwide [1], with knee OA (KOA) exhibiting an incidence of 240 per 100,000 person-years in adults or about 2.5 times that of hip OA [2].

Further known or accepted, governmental-approved guidelines for management in primary care in Denmark and Sweden state that a KOA diagnosis can be made clinically and that strength training and education are among the first-line of care [3]. Diagnostically, this concurs with the criteria of the European League Against Rheumatism’s (EULAR) [4] for primary care, on the one hand. For hospital care, on the other hand, much accepted criteria are those from the American College of Rheumatology (ACR) [5]. More recently, KOA is conceptualized as a whole person chronic disease [6], one where symptoms and signs develop over decades [7], and that is manageable for most people by early diagnosis and individualized management [6].

Management by strength exercise therapy demonstrates the largest effect sizes (ES) on pain and function compared to other active therapies in KOA according to systematic reviews and meta-analyses of randomized controlled trials (RCTs) [8–11]. Further, in the most affected leg, systematic reviews of cross-sectional case-control studies indicate muscle weaknesses across the hip [12] and the knee muscles [13] for patients with KOA. However, even for the affected leg, there is limited knowledge of *the relative difference* between ankle, knee, and hip muscle strength in patients with KOA compared to healthy controls. Furthermore, there is particularly limited knowledge regarding ankle strength. Additionally, regarding the (least or) non-affected leg, knowledge about strength deficits and their relative difference is even more limited. Overall, such extensive strength knowledge can potentially lead to improved strategies for strength exercises for patients with KOA.

Thus, to improve the knowledge required for strength exercise therapy, we aimed to explore the relative difference in muscle strength bilaterally in the main directions across the hip, knee, and ankle joints between patients with KOA and individuals without knee complaints in a cross-sectional study.

More specifically, by application of traceable and reliable strength dynamometers, we performed the first ever full bilateral overview of strength deficits of the main muscle-groups of the lower limb in patients with KOA compared to healthy controls through a well-powered exploratory study [14, 15].

## Methods

This study was a part of a larger comprehensive study of functional aspects on knee osteoarthritis, FUNKART.

### Design and ethics

We set out to develop an exploratory [14, 15] cross-sectional, age- and gender-matched [16] patient versus healthy control study. The study was approved by a Regional Ethics Committee for Medical and Health Research (the Regional Ethics Committee North, REC-north 2016/984) and conducted according to the Helsinki declaration and Norwegian laws. All participants received oral and written information and signed an informed consent before entering the study.

We recruited individuals with KOA referred by general physicians (GPs) to private physiotherapy clinics and to the osteoarthritis school at a university hospital from Nov 2016 to Dec 2017. Frequency-matched [16] healthy volunteers were aimed to be recruited from work places in the vicinity of the lab by in-person, physical and electronic communication.

### Participants

The inclusion criteria for patients were having KOA in the tibiofemoral joint (s) of one or both legs diagnosed clinically and verified radiologically (Kellgren-Lawrence grade 1–4) [17], with main problem of pain and limited physical function related to the knee (s) and be symptomatic for >3 months and daily during last month.

The inclusion criteria for both groups were being male or female between 45 and 70 years of age, able to understand written and oral Norwegian, and be able to walk on even ground and stairs. Healthy controls had to be without pain or knee complaints during common activities of daily life.

The exclusion criteria for all participants were surgery to a lower extremity < 3 years ago, prior lower limb fractures, generalized pain, pain from the spine, hips, or ankles competing with that from the knee (in the KOA-group), knee pain (in the control-group), body mass index >35, and medical diagnoses other than KOA with clear negative influence on physical function and pain.

### Measurements

#### Strength measurements

Before strength testing, the participants warmed up by several performance tests (e.g. the 6-min walk test [6MWT] and the 10-step up-and-down stair-climb test [T10StUpDwT]) and a set of 15 repetitions at low-to-moderate load on each specific exercise. Strength or concentric peak torque were recorded at 60°/s by the isokinetic mode applying the Biodex® System 4 Dynamometer (Biodex Medical Systems, NY, USA) [18, 19]. With the participants sitting, we sequentially tested strength with the back rest tilted 70° off the horizontal line in the following order: knee flexion and extension, hip internal and external rotation, ankle inversion and eversion, and ankle plantar and dorsal flexion. With the participants supine, we tested hip extension and flexion. These positions and setups were according to the Biodex manual [20], except for the hip rotation tests that were performed according to Baldon et al. [21]. Finally, with the participants supine on a therapy bench, we tested isometric hip abduction and adduction strength with the hips in a neutral ab-adducted position, applying a hand-held muscle tester dynamometer (HHD) according to Thorborg et al. (2013) [22]. Specifically, the HHD (Commander Muscle Tester, JTech Medical Industries, Utah, USA) was placed under a non-elastic fixation belt (art. no. 304018, Fysiopartner, Norway) that was looped around the epicondyle of the femur and a vacuum pump [that was fastened on the wall] (art. no. 071458045, Würt, Germany). Before testing, the pelvis was secured bilaterally against inferior and lateral displacement according to Vaarbakken and Ljunggren [2007] [23].

We applied five consecutive maximum strength tests by the Biodex system and three repeated trials by the belt-fixed HHD. Oral encouragements were applied according to principles in Thorborg (2013) [22]. For the knee tests, the Biodex system’s “passive isokinetic mode” was chosen, to better accommodate eccentric performance (eccentric data not reported here). Accordingly, fully passive recordings were taken to correct for gravity (see Data processing). The other tests by the Biodex system, we corrected for gravity by its software. The Biodex system was calibrated before each session according to the manual [20]. The HHD is certified by the National Institute of Standards and Technology (NIST) standards. The latter device is self-calibrating and was compared daily to an identical reserve HHD, as both were compared to traceable Olympic Competition Weights [24] (Eleiko, Halmstad, Sweden). The test team trained about 40 h to execute the complete strength protocol within 1.5 h.

### Procedures and supplementary measures

To enable appropriately judging the background variables (demographics, personal, and clinical factors) and the warm up procedures of the present study, we present below supplementary measures mainly presented in Table 1 (Results) and another study in the present journal [25].

**Table 1:** Background or personal and clinical factors in the case- and control-group

ICF	Variables	Cases (*n* = 28)	Controls (*n* = 31)	Statistics t, χ2, U	*P*-value
Personal factors	Female, n (%)	18 (64)	16 (52)	379 (χ2)	0.3294
	**Age, yrs., M (SD)**	**61.7 (6.4)**	**55.3 (8.0)**	**3.4 (t)**	0.0014*
	Height, m, M (SD)	1.72 (0.10)	1.73 (0.09)	-0.7 (t)	0.517
	Weight, kg, M (SD)	82.9 (12.7)	80.4 (16.6)	0.7 (t)	0.517
	BMI, kg/m^2^, M (SD)	24.3 (3.5)	25.2 (5.1)	1.0 (t)	0.317
	Education, n (%)				
	secondary school (10 yrs)	1 (4)	0 (0)		
	high school (13 yrs)	6 (21)	6 (19)		
	graduate (16 yrs)	14 (50)	13 (42)		
	post graduate (18 yrs. +)	7 (25)	12 (39)	368 (U)	0.281
	Dominant leg (right, left, n)	26, 2	28, 3		
Body function	Years since diagnosis, M (SD)	10.2 (8.6)			
	Years of knee pain, n (%)				
	1 yrs	2 (7)			
	1 to 3 yrs.	3 (11)			
	3 to 10 yrs	7 (25)			
	> 10 yrs	16 (57)			
	Pain last week, Med (IQR)	3.5 (4.8)	0.0 (1.0)	3.0	
	KOOS Pain, Med (IQR) (R)	58.8 (18.8)	98.4 (3.6)	−38.9	
	**Case-group only**				
	X-ray grade (n knees, %)	Inv. leg	Uninv. leg		
	No X-rays taken	0 (0)	10 (36)		
	KL-grade II	9 (32)	9 (32)		
	KL-grade III	17(61)	8 (29)		
	KL-grade IV	2 (7)	1 (4)		
	KOOS Sympt., Med (IQR) (R)	58.9 (33.9)	96.4 (7.1)	−35.8	
Activity function.	KOOS ADL, Med (IQR) (R)	66.7 (39.6)	100.0 (13.3)	−32.4	
	KOOS Sport/Rec, Med (IQR) (R)	30.0 (25.6)	100.0 (5.0)	−65.0	
Participation function	KOOS QoL, Med (IQR) (R)	43.8 (25.0)	100 (13.9)	−56.2	

Notes: Bold font and * = highly significant different variable; *KOOS* Knee Injury and Osteoarthritis Outcome Scale, 0 to 100, worst to best; Sympt. = symptoms (a KOOS subscale); *ADL* Activity in daily life, Sports/Rec = Sports and Recreation; t = Independent t-test statistics; χ2 = Chi-square test statistics; U = Mann-Whitney U-test statistics. KL-grade: Kellgren-Lawrence osteoarthritis grade

For each participant, questionnaire, functional, and strength data were collected within a period of 2 weeks. The questionnaires were e-mailed as web-surveys together with the informed consent forms by the Infopad system [26]. All participants filled out the self-reported outcome measurement instruments Numeric Pain Rating Scale (NPRS) [27–29] and Knee Injury and Osteoarthritis Outcome Scale (KOOS) [30–32]. KOOS was chosen (over the more widely applied Western Ontario and McMaster Universities Arthritis Index [WOMAC]) due to being free/open access [33], its inclusion of Recreation and Sports and WOMAC, and its knee specificity. In the week thereafter, in the lab, we registered personal or demographic characteristics, degree of radiographic KOA (radiology reports) [17], the 6MWT [34, 35], and the T10StUpDwT [33, 34, 36]. (The latter tests were embedded as a strength warm-up procedure and their results are reported elsewhere [25].) At the end of the lab-session, we measured peak strength. In all the tests, the Biodex “cushion -function” was set at hard and the windowing to 80%. The study’s questionnaires took on average 40 min and the total test protocol 2.7 h (that is, in the extended or full study protocol).

### Data processing

For concentric knee *extensor* strength, the passive torques were added to the active ones to correct for the limb’s own torque, whereas for concentric knee *flexion* strength the passive torques were subtracted. Peak knee strength at the 30° knee flexed position (0° = straight knee) was reported. The isokinetic mode with automatic gravity correction was used for all but the knee flexion and extension protocol in passive mode where gravity was corrected for afterwards.

For the HHD isometric hip tests, we calculated torque (Nm) by multiplying force (N) by the distance (m) from the top of trochanter major to the femoral lateral condyle. Strength was taken as “best of” or peak torque and divided by body weight, thus we report Nm/kg.

### Statistical analysis

A pilot-based sample size calculation, based on unpublished lab-data from an osteoarthritis study in the same area [37], with α (two-tailed) of 0.05, β 0.20, SD 0.7, and moderate effect size of 0.64, gave the estimate that 20 participants was needed per group. However, due to the explorative design [15], we aimed for 30 participants in each group.

Normality was inferred by histogram inspections, Normal P-P plots, and Kolmogorov-Smirnov tests. For the equal variance assumption, Levene’s test were performed. For parametric strength data with no significant outliers and equal variance, a one-factor (lower limb test: *n* = 12 strength tests) two level (left and right sides) full factorial (including side × group interaction) repeated measures multiple analysis of covariance (repeated measures MANCOVA), with age as covariate, were performed. This was performed to evaluate the overall effect of group for strength across the whole kinetic chain for both sides. Effect sizes as eta square (η^2^), were interpreted according to Cohen [38] as low <0.04, medium ≥0.04 to <0.36, and large ≥0.36.

Secondly, as a post hoc test for comparing *between-group differences* for each side, an independent measures analysis of covariance [ANCOVA] (most involved vs non-dominant side, least involved vs dominant side) was performed, with age as the covariate. Based on the age-adjusted means, we calculated adjusted standardized mean difference (SMD) or effect sizes (ES) by Cohen’s *d* [38] with 95% CI [39], where 0.2, 0.5 and 0.8 were considered small, moderate and large, respectively. Personal or demographic variables were compared between groups with unadjusted and independent univariate conventional statistics (see Table 1, Results). Alpha was set to 0.05 for all statistical tests (SPSS, v.25, IBM, NY, USA), as no adjustments are needed for multiple comparisons in explorative studies [14–16, 40–42]*.*

## Results

### Recruitment result

Two patients were recruited in physiotherapy clinics, without information on those who declined. At the hospital, we recruited 27 patients out of 36 eligible, where 10 of those invited chose not to participate. The reasons for declining were long traveling distances (*n* = 3), not interested (*n* = 4), afraid of strength testing (*n* = 2), and too time-consuming (*n* = 1). One participant answered the questionnaire but withdrew before the lab-test due to a flare-up at home. This person later withdrew with no stated reason and was excluded from the analysis. Five individuals with KOA were excluded from participation due to old age (*n* = 3), BMI, and an unstable heart. Thus, in total we analyzed/included 28 patients and 31 healthy controls in this study.

We aimed to match the groups on gender and age, however, because of inadequate recruitment in the private physiotherapy setting, we had to change the recruitment into secondary/hospital care with comparably older patients. Then, as the recruitment of healthy subjects was aimed at the working population (due to problems with recruiting unaffiliated healthy early pensioners), we experienced a between-group difference in age that we had to adjust statistically.

### Personal and clinical characteristics

The patients with KOA were on average 6.4 years older (than the controls). There were no other significant differences for personal/demographic factors. On average, the patients had experienced pain for 11 years, and they were diagnosed 10 years ago. Further, they had mostly small to moderate radiographic KOA and moderate symptoms. Table 1 shows the personal/demographic and non-strength clinical factors.

A significant moderate *overall* interaction effect of side and group was indicated when all measures for both sides were collapsed into the repeated measures MANCOVA model (Wilks’ Lambda [WL] = 0.825, F_1, 56_ = 11.845, η^2^ = 0.175, *P* = 0.001). However, then there was no significant main effect for side (WL = 0.997, F_1, 56_ = 0.161, η^2^ = 0.003, *P* = 0.690) nor an interaction effect between side and age (WL = 0.998, F_1, 56_ = 0.119, η^2^ = 0.002, *P* = 0.731).

A main effect of group showed a near significant difference in total strength for the whole kinetic chain and both sides (F_1, 56_ = 3.902, η^2^ = 0.065, *P* = 0.053) indicating a strength deficit in the KOA group.

The post hoc test *between-group* ANCOVA, however, showed significant muscle weakness of moderate magnitude in six joint–strength directions on the (most) involved side in patients with KOA compared to controls. Specifically, the most substantial muscle weaknesses were found in hip internal rotation and ankle eversion, and hip external rotation. Further, still moderate but somewhat smaller weaknesses were found in knee extension and ankle dorsal flexion and inversion on the most involved side. The only significant weakness finding on the (least or) uninvolved leg was for ankle dorsal flexion (Table 2). There were no significant differences between groups for 17 of the 24 strength tests.

**Table 2:** Strength differences between patients with KOA and control individuals without knee complaints unadjusted and adjusted for age

	Strength Joint Dir	KOA-group		HC-group		SMD		KOA – HC group		
R_n_		M (SD) [in Nm/kg unit]						M % d	*P*-values	
		Unadj (SD)	Adj M (SD)	Unadj M (SD)	Adj	Unadj	Adj (95% CI)	Adj	Unadj	Adj
1	**Hip IR I**	0.74 (0.27)	0.75 (0.25)	0.96 (0.21)	0.94 (0.25)	−0.9	−0.7 (−1.3, − 0.2)	22.5	0.0009	**0.0092†**
2	**Ankle EV I**	0.19 (0.08)	0.20 (0.08)	0.27 (0.07)	0.26 (0.08)	−1.0	−0.7 (− 1.3, − 0.2)	26.1	0.0006	**0.0096†**
3	**Hip ER I**	0.25 (0.10)	0.26 (0.12)	0.36 (0.13)	0.35 (0.12)	−0.9	−0.7 (−1.2, − 0.2)	29.5	0.0008	0.013*
4	**Knee EXT I**	1.16 (0.48)	1.15 (0.46)	1.46 (0.38)	1.48 (0.46)	−0.7	− 0.7 (−1.2, − 0.2)	25.1	0.0103	0.012*
5	Ankle PF I	0.56 (0.24)	0.58 (0.25)	0.75 (0.23)	0.73 (0.25)	−0.5	− 0.6 (−1.2, − 0.1)	22.9	0.0029	0.073
6	Ankle DF I	0.17 (0.07)	0.17 (0.08)	0.23 (0.09)	0.22 (0.08)	−0.8	−0.6 (−1.1, − 0.1)	25.6	0.0043	0.025*
7	Ankle INV I	0.24 (0.10)	0.25 (0.10)	0.31 (0.08)	0.31 (0.10)	−0.8	−0.6 (−1.1, − 0.1)	21.4	0.0055	0.033*
8	Hip AD U	0.75 (0.33)	0.76 (0.31)	0.93 (0.25)	0.92 (0.31)	−0.6	−0.5 (−1.0, 0.0)	19.0	0.0227	0.057
9	**Ankle DF U**	0.17 (0.06)	0.18 (0.08)	0.23 (0.08)	0.22 (0.08)	−0.7	− 0.5 (−1.0, 0.0)	20.0	0.0096	0.021*
10	Hip AD I	0.71 (0.27)	0.72 (0.27)	0.86 (0.24)	0.85 (0.27)	−0.6	− 0.5 (−1.0, 0.0)	16.6	0.0272	0.057
11	Knee FLX I	0.59 (0.35)	0.61 (0.33)	0.77 (0.28)	0.76 (0.0.33)	−0.6	−0.5 (−1.0, 0.1)	21.9	0.0325	0.114
12	Hip FLX I	1.15 (0.29)	1.15 (0.32)	1.29 (0.31)	1.29 (0.32)	−0.4	− 0.4 (−1.0, 0.1)	11.5	0.0749	0.256
13	Knee EXT U	1.45 (0.36)	1.45 (0.44)	1.64 (0.46)	1.64 (0.44)	−0.5	− 0.4 (− 0.9, 0.1)	11.6	0.0877	0.120
14	Hip EXT I	1.20 (0.52)	1.21 (0.52)	1.44 (0.45)	1.43 (0.51)	−0.5	−0.4 (− 0.9, 0.1)	16.7	0.0701	0.135
15	Hip AB U	0.85 (0.37)	0.88 (0.36)	1.05 (0.33)	1.02 (0.36)	−0.6	−0.4 (− 0.9, − 0.1)	14.7	0.035	0.148
16	Ankle PF U	0.59 (0.27)	0.61 (0.27)	0.73 (0.26)	0.71 (0.27)	−0.6	−0.4 (− 0.9, 0.1)	15.2	0.0379	0.185
17	Hip FLX U	1.19 (0.30)	1.19 (0.35)	1.30 (1.30)	1.30 (0.35)	−0.3	−0.3 (− 0.8, 0.2)	8.8	0.2043	0.105
18	Hip ER U	0.29 (0.11)	0.30 (0.12)	0.34 (0.13)	0.33 (0.12)	−0.5	−0.3 (− 0.8, 0.2)	9.5	0.0671	0.288
19	Hip AB I	0.90 (0.35)	0.91 (0.34)	1.01 (0.29)	1.00 (0.34)	−0.4	−0.3 (− 0.8, 0.2)	9.4	0.19092	0.148
20	Ankle EV U	0.23 (0.08)	0.24 (0.09)	0.26 (0.08)	0.26 (0.08)	−0.5	−0.2 (− 0.8, 0.3)	8.0	0.0911	0.375
21	Hip EXT U	1.31 (0.51)	1.34 (0.54)	1.50 (0.51)	1.47 (0.54)	−0.4	−0.2 (− 0.8, 0.3)	9.3	0.1499	0.146
22	Knee FLX U	0.74 (0.38)	0.77 (0.39)	0.90 (0.37)	0.87 (0.39)	−0.4	−0.2 (− 0.7, 0.3)	12.2	0.1042	0.390
23	Ankle INV U	0.25 (0.09)	0.26 (0.08)	0.28 (0.07)	0.27 (0.08)	−0.3	−0.2 (− 0.7, 0.3)	3.8	0.2252	0.534
24	Hip IR U	0.80 (0.24)	0.82 (0.24)	0.87 (0.21)	0.85 (0.24)	−0.3	−0.1 (− 0.7, 0.4)	3.6	0.2768	0.588

Notes. Statistically significant differences are in bold type. *KOA* Patients in the knee osteoarthritis group, *HC* Healthy control group, *R*_*n*_ Rank-position for the joint-and-torque-direction on muscle weakness (i.e., SMD) in the KOA-group compared to the HC-group, * = significant different, † = highly significant (two-tailed ANCOVA); *Unadj* Unadjusted, *Adj* Adjusted for the covariate age, *I* the (most) involved leg (in KOA-group) or non-dominant leg (in HC-group), *U* Uninvolved leg or dominant leg (if HC-group), dir. = direction, *EV* Eversion, *ER* External rotation, *IR* Internal rotation, *INV* Inversion, *DF* Dorsal flexion, *EXT* Extension, *FLX* Flexion, *PF* Plantar flexion, *ER* External rotation, *M* Mean, *SD* Standard deviation, *CI* Confidence interval (lower limit, upper limit), *SMD* Standardized mean difference or Cohen’s d, *p P*-value. All strength measures are peak strength regardless of range of motion, except for the knee joint (peak strength at 30° flexion) and hip AB or AD (peak strength in the anatomical position). Results are normalized for body mass (i.e., M and SD are given as Nm/kg)

**Fig. 1:**
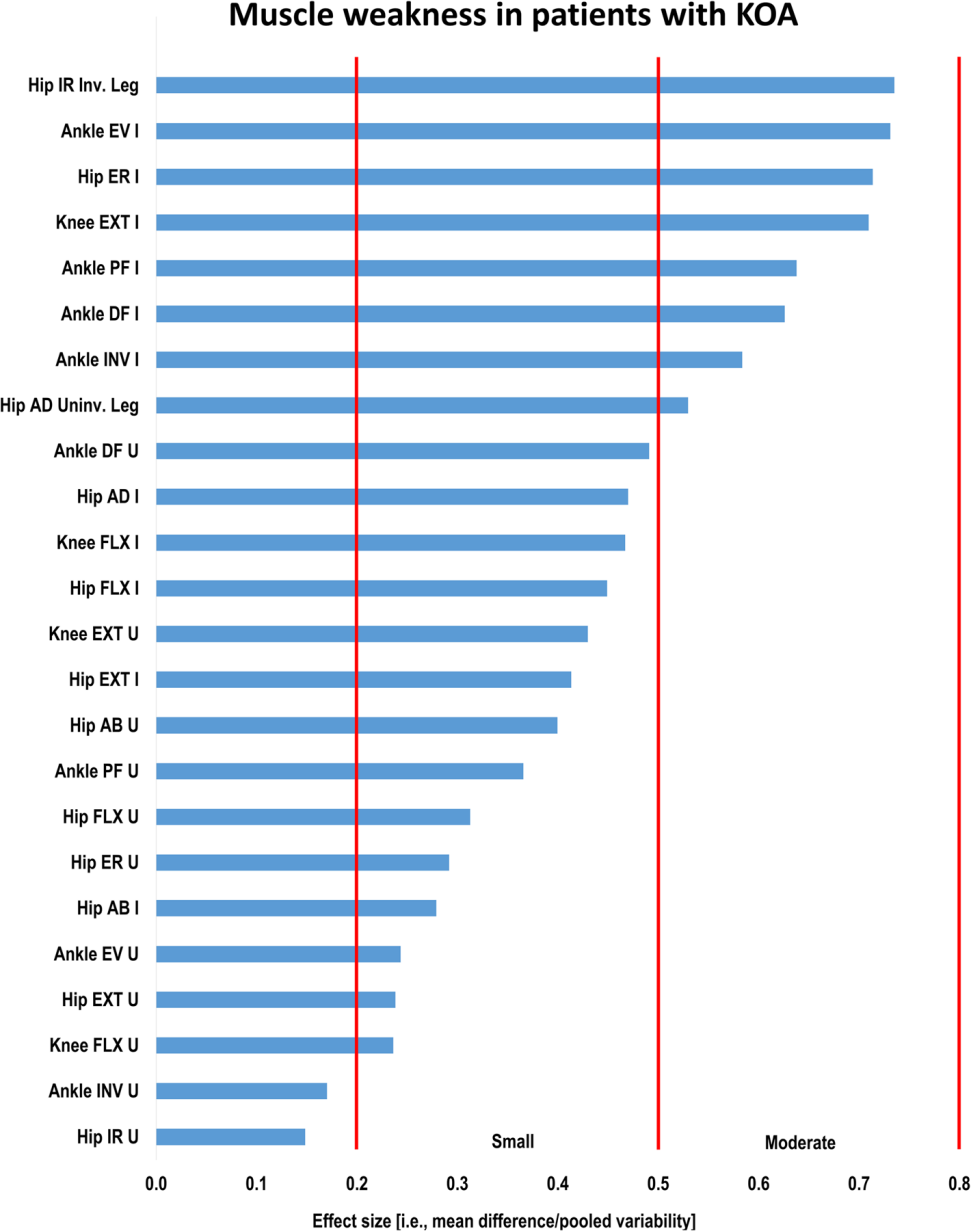
Muscle weakness as difference between patients with knee osteoarthritis compared to individuals without knee complaints. Strength directions for joints with largest weaknesses on top and the smallest on the bottom. Notes: Effect size = Standardized mean difference or Cohen’s *d*, KOA = knee osteoarthritis, Inv. = (most) involved leg, I = (most) involved leg, EV = eversion, ER = external rotation, FLX = flexion, EXT = extension, PF = plantar flexion, DF = dorsal flexion, INV = inversion, AD = adduction, Uninv. = (least or) uninvolved leg, U = (least or) uninvolved leg, AB = abduction

Figure 1 indicates the relative difference in muscle weakness across legs and joint–strength directions in the patients with KOA compared to healthy individuals.

## Discussion

### Principle findings

Overall, across legs and join–strength directions, the most substantial muscle weakness were found in the involved leg for the muscles that evert and invert the ankle (i.e., that effect mainly frontal-plane shank-foot-ground interactions during gait) and for muscles that internally and externally rotate the femur at the hip (i.e., that effect mainly transversal-plane pelvic-femur interactions). Still further on the involved side, we found about the same magnitude of weakness for the muscles that extend the knee and plantar and dorsal flex the ankle (i.e., that effect mainly sagittal-plane femoral-shank-foot-ground interactions). There were no significant differences for the remaining 67% of the tests. This is the first study that has comprehensively explored muscle strength across the whole kinetic chain of the lower extremities bilaterally in patients with KOA versus healthy controls.

### Results discussion

The current finding of most substantial hip external rotation weakness, is fairly concurrent with three other case-control studies [43–45]. Our ad hoc meta-analysis of these (studies) showed a large between-group difference (30% in mean; ES 0.9, 95% CI 0.4 to 1.37) based on 163 cases and 97 controls. Further, their gender distributions agreed with that seen in our study. Because there are now three studies with similar point estimated and variable discriminative findings, adding more studies with the same small-to-moderate severely affected KOA population might not change this evidence. As for clinical trial evidence, a recent systematic review [11] of randomized control trials (RCTs) demonstrated large effects on pain and function of hip strengthening exercises and quadriceps exercises as compared to quadriceps exercises alone. Unfortunately, the methodological quality of these trials was low (i.e., a median PEDro score < 6). Interestingly though, none of these trials reported to have strengthened the hip external rotators, only the hip abductors. However, note that the hip abductor exercises in these trials probably indirectly exercised four out of 13 muscles known to externally rotate the hip [46]. Thus, in sum, evidence indicate substantial discriminative value of assessing external rotation strength with a promising but insecure and indirect link to strength exercise therapy improving pain and function for patients with KOA.

Hip internal rotation weakness was reported in two previous case-control studies [43, 44], a between-group difference documented in a meta-analysis [12] to be large (29% in mean; ES 0.8, 95% CI 0.3 to 1.2). That result [12] is in fair agreement with the current study through various differences: The proportion of females [88% [44], ours 62%], not reported BMI [43] and higher BMI [44], various positions of measuring, and measurement modes [isometric [43], isokinetic 30°/s [44], ours isokinetic 60°/s]. As for clinical effects, however, through two recent systematic reviews [11, 47] we found no trials that had specifically targeted the hip internal rotators. However, on pain and function, these reviews showed important indirect effects of exercising the hip internal rotators by using programs that applied hip abductor exercises which probably indirectly loaded three out of seven hip internal rotators [46]. Thus, evidence indicate important test discrimination and indirect exercise effect of hip internal rotation strengthening on pain and function in KOA.

Ankle strength is the least examined construct as compared to studies on knee and hip strength. On the one hand, we did not find other case-control data on *ankle eversion* strength. Such strength is also unreported for healthy individuals according to a recent systematic review [48]. On the other hand, the *ankle inversion* muscle weakness in the current study is slightly less pronounced than the finding of Park et al. (2016) [45], whom reported a large effect size (0.84, 95% CI 0.25 to 1.43) of isometric testing presented as N/kg (vs ours Nm/kg). Strength, however, is most validly presented as Nm/kg [49]. A more important risk of bias in that study [45] appears to be the lack of reporting the exact method of measuring inversion strength. Thus, the above wide confidence interval, low number of studies, and the methodological uncertainty, makes this evidence very likely to change with future studies. As for clinical trial effects, we found no prior strength exercise studies having explicitly reported targeting these mainly frontal plane ankle muscles. Thus, in sum, evidence indicates uncertain but substantial discrimination on ankle strength mainly in the frontal plane with an unexplored therapeutic link in KOA.

The knee extension weakness in patients with KOA is large on average. According to a recent meta-analysis of 27 cross-sectional case-control studies [13] whereto we added five more [45, 50–53], the between-group difference amounted 23% and a large effect size (0.8, 95% CI 0.2 to 1.5). The present study’s moderate muscle weakness thus falls into the middle to lower range of this confidence interval. Possibly the muscle weakness in the current study could have been more pronounced if our data had been extracted in a more flexed knee position than 30°. Indeed, among the 11 highest ranked studies in our ad hoc meta-analysis, we found large knee extension weakness among all five isokinetic studies [54–58] that recorded peak strength at 54° of knee flexion on average (our calculation). The large muscle weakness variability in the total meta-analyzed result and the small lower limit of its confidence interval, indicate that the true knee extension weakness does indeed vary largely in this population, a fact that is unlikely to change with future research. On pain and function, the clinical importance of knee extension strength exercises in KOA is indisputable [8, 59].

The current study found moderate weakness in ankle plantar flexion. Previous case-control results were meta-analyzed [45, 50, 52, 60–63] and showed large difference (24% in mean; ES 0.82, 95% CI 0.3 to 1.3) between 301 cases and 272 controls. Again, this muscle weakness is more substantial than our moderate finding and those studies represent a lower proportion of females (38% females vs. our 64%). Further, the mean difference in percentage from the meta-analyzed material ranged from 50 to 1% (vs ours 19%) and confidence intervals ranged from small to large. Thus, this evidence is likely to change with future studies, although it might as well indicate a truly large sample variation. Of promising therapeutic importance, the plantar and invertor muscles have indicated a substantial external knee abduction moment via their impact on the ground reaction force during gait [64]. This seems important, due to its possible mitigating effect on a highly prevalent medial radiographic KOA shown to be positively associated with (although unproven to be caused by) an increase in the external knee adduction moment [65]. However, on pain and function, the only evidence of therapeutic effects of ankle plantar flexion exercise appears to be indirect; that is, through trial programs strengthening the kinetic chain through the one-legged press only [66, 67]. Thus, evidence indicate substantial point discrimination and variability of assessment and indirect exercise effects on pain and function of ankle plantar flexors strength in KOA.

The biomechanical mechanisms of KOA appear to be knee instability and muscle weakness in the frontal [51, 68], transverse [64, 69], and sagittal plane [13]. That is, the mechanisms behind the long-term symptomatic KOA might be selective weakness of the soleus and gastrochnemius [64], the fibularii, the tibialis anterior, the hip internal-external rotators, and the quadriceps muscle [actuating sagittal and frontal plane control [70]]. Here we present recent arguments, starting off in the frontal plane.

A particularly strong cross-sectional case-control study [51] indicate joint instability in the frontal plane and thereto cartilage wear as a plausible injury mechanism. Having applied highly accurate dynamic stereo X-rays and instrumented gait-way analyses in patients with medial KOA, Farrokhi et al. (2016) [51] found significant (i) elevated tibiofemoral contact point excursions and (ii) elevated frontal plane motion, both during the loading response phase of downhill gait. Further, a case-control simulation study based on in vivo biomechanical analysis of horizontal gait in patients with varus misaligned KOA [64], indicated that the soleus and gastrocnemius muscles offered a significant deficit in external knee abduction moment (effected actively via the ground reaction force) in patients with KOA. That is, a deficit capable of explaining the patients’ increased external knee adduction moment at its second peak during late stance phase. This second peak was three times higher than that in the control individuals as compared to the first peak (that was mainly caused by gravity). In the same study [64], gluteus medius was the primary contributor to the external knee adduction moment (via the ground reaction force) in both cases and controls (i.e., a normal finding). However, a major limitation of their [64] muscle modelling was not having included the large gluteal muscles as knee-spanning muscles (i.e., the tensor fascia lata, gluteus medius, and gluteus maximus via their common long tendon - the fascia lata/iliotibial band) [71, 72]. The knee-spanning gluteals probably contribute substantially to the *internal* (i.e., possibly protective) knee abductor moment due to its large cross-sectional area, long tendon, and large moment arm (as compared to that of the quadriceps in the frontal plane [70]). Comparingly, when preparing for the present study, we found no reliable test for knee abduction strength. Further, the tests found reliable for hip abductor strength in patients with KOA didn’t apply resistance inferior to the knee joint, and therefore did not include any knee-spanning moment of the gluteal knee abductor muscles.

Further in the frontal plane, a prospective cohort study [68] biomechanically assessed patients with varus mal-aligned KOA during gait. Here, Hodges et al. (2016) [68] documented positive correlation between annual loss of medial tibial cartilage volume and (i) greater duration of medial knee muscle (vastus, semimembranosus) co-activation, and (ii) greater duration of medial relative to lateral knee muscle (vastus lateralis, biceps femoris) co-activation. *Higher* lateral thigh-muscle co-contraction correlated significantly with *decreased* cartilage loss. A possible explanation for these patients’ apparent mal-adaptive increase in muscular compression across the medial tibiofemoral joint, is that these medial knee-spanning muscles are capable of increasing the external knee abduction moment via their (joint-coupled) influence on the ground reaction force [64].

In the transverse plane, in downhill walking – the most problematic activity for patients with KOA [51] – most of the deep external rotators of the hip are at short length and thus force–length weakened (due to the slightly flexed-to-extended positions of the hip) [73, 74]. That is, the already weakened external rotators, as tested in lengthened positions in the present study, become even weaker by the downhill-walking hip movement pattern. Further, the external rotators of the hip are documented as the group most vulnerable to muscle weakness during gait [75]. As for the role of muscle weakness of the hip internal rotators, however, we speculate that they have an important co-contracting and hip-stabilizing role in concert with the external rotators, much similarly to that of the hamstring muscles concerting the main knee muscle quadriceps during external knee flexion moment loading in the sagittal plane [70].

Thus, in support of (i) the present study, (ii) strength trial meta-analyses [12, 13], and (iii) in-vivo anchored simulations [64], possible therapeutic solutions might be as follows: To increase the strength of the hip external and internal rotators and knee-spanning hip abductors, the lateral knee extensors and flexors (i.e., the knee-spanning knee abductors), and the ankle invertors and plantar flexors (i.e., the non-knee spanning knee abductors). On the core outcomes pain and function, evidence from two systematic reviews of randomized controlled trials [2018] [11, 47] evaluating the effect of hip muscle strength exercises [47], and hip muscle strength exercises in addition to knee extension strength exercises [11], indirectly hints towards such a mechanism in patients with KOA.

### Methods discussion

The current study has its methodological limitations and strengths. On the one hand, we did not manage to level the groups equally on age, and some readers might miss an alpha correction for the multiplicity of testing according to classical statistical texts [76–80]. Further, the results of the peak knee extensor and flexor strength were confined to the 30° knee position, and the sample size was moderate [76, 77]. Moreover, there is evidence of relation between reduced strength with increasing radiographic KL-grade of KOA [81] unadjusted for in the present study. Yet further, one may claim that these strength differences are due to malalignment [82]. Finally, one can ask: could not all the current muscle weaknesses be explained by pain [83–86]? On the other hand, this is the only study so far to have comprehensively explored muscle strength in all main joints and directions bilaterally in a single case-control sample for patients with KOA. Further, we statistically adjusted for the difference in age. Supportingly therein, there was no substantive difference in the statistical inferences between the age-adjusted and the unadjusted analysis. The latter fact is understandable, due to the mean in groups being within the same middle-aged maturational category [45–64 years old] (MeSH, PubMed). Thus (therein), the groups were presenting themselves with the similar age-vs-strength decline risk profile. Concordantly, our findings (adjusted or unadjusted) were well aligned with those from appropriately age-matched confirmatory case-control studies. Indeed, in the present study we generally found *less* pronounced between-group differences than what was found in prior studies summarized in meta-analyses thus contradicting an alleged age bias. Further, the explorative nature of this study justifies its main findings by highly significant differences, and corrections for multiple comparisons are judged by reputable statisticians not to be needed in exploratory studies [14–16, 40–42]. Yet further, our peak knee extension strength position of 30° adds valuable data compared to the average peak strength position of 54° of prior isokinetic case-control studies [13]. Moreover, there is way more evidence *against* an association between radiographic grade of KOA and strength [82, 87, 88] than the indirect association found *for* it in a single cohort [81]. Yet further, there is systematic review and meta-analysis evidence against the association between KOA and malalignment [13]. Even further, although several studies show an association between increased pain and decreased strength (chiefly in the knee extensor muscles), there exists opposing evidence [83, 89–91]. More importantly thereto, the current study was not designed to build a strong presumably causative or associative claim as to *why* these patients were weaker in all these muscle-groups. Thus, we infer adequate internal validity of the current study.

The extensiveness of our testing of muscle groups in the lower limb is limited by excluding the toe flexor muscles [92–94]. Additionally, the external validity of the study is limited to patients below 70 years of age and BMI obesity class I (excluding WHO’s obesity grade II-III). Furthermore, because the current sample size was moderate and the study exploratory designed [14, 15], we acknowledge the need for larger exploratory and confirmatory studies to further substantiate the present findings. Still, we infer the current study to be appropriately externally valid.

### Potential clinical research implications

What might be the possible clinical research implications of the evidence analysis above? In order to improve pain and function, clinical researchers may apply ours and others’ case-control findings, together with meta-analytic trial evidence [11, 12], to incorporate strengthening of weak ankle and hip muscles into the existing so called “hip abductor exercises” [95, 96] together with a simple and effective [97] open chain quadriceps program [98]. Then all this can be compared to a control group given the latter active quadriceps exercise program [98] only. The first protocol is hypothesized to account for the possibility that the most important muscles for an apparent knee cartilage protecting internal knee abduction moment [65, 68] might be the quadriceps [70] and the knee-spanning gluteal muscles [64, 71, 99]. Interestingly, these latter knee-spanning gluteals, together with the hip external rotators [73] and the ankle evertors, are probably all strengthened in the promising *standing hip-flexed wall abduction exercise* described in Ashok’s recent RCT [95]. Interesting indeed, because, according to a systematic review and meta-analysis of RCTs [11], that particular exercise is described in the most effective experimental program on pain and function as compared to an active quadriceps control-exercise group in the Ashok (2012) trial [95].

## Conclusions

Conclusively, this exploratory study indicates that the most substantial muscle weaknesses are in the involved leg’s hip and ankle muscles with main actions in the frontal and transverse planes of the kinetic chain of importance for gait. Slightly less substantial, it still indicates important weakness of the knee extensor muscles. That is, in patients aged 45 to 70 years with knee osteoarthritis with light-to-moderate disease severity in a primary/hospital care setting. Future confirmative studies are needed to evaluate the validity and clinical relevance of these findings. Clinical trialists are suggested to build on existing strength programs that already include these ankle and hip muscle-groups in addition to the knee extensor muscles, and that appear highly effective on pain and function according to a meta-analysis of randomized controlled trials.

## Data Availability

The dataset generated and analyzed during the current study are available on reasonable request from the head of the project AKS or from the Department of Neuroscience and Movement Science, Faculty of Medicine and Health Science, Norwegian University of Science and Technology.

## Abbreviations

6MWT: Six-minute walk distance test
ACR: American College of Rheumatology
ANCOVA: Analysis of covariance
BMI: Body mass index
CI: Confidence interval
ES: Effect size
EULAR: European League Against Rheumatism
FUNKART: the mechanism for function with knee osteoarthritis study
GP: General physician
HHD: Hand held dynamometer
KOA: Knee osteoarthritis
KOOS: Knee Injury and Osteoarthritis Outcome Score
MANCOVA: Multiple analysis of covariance
N: Newton
NIST: National Institute of Standards and Technology
Nm: Newton meter
OA: Osteoarthritis
PEDro: Physiotherapy Evidence Database
P-P plots: Probability-probability plot
RCT: Randomized controlled trial
REC: Regional Ethics Committee
SMD: Standardized mean difference
T10StUpDwT: Timed 10-Step Up and Down Stair Climb Test
